# Utilidad clínica de la biopsia líquida para el diagnóstico y seguimiento de los pacientes con CPNM y *EML4-ALK*

**DOI:** 10.1515/almed-2020-0007

**Published:** 2020-03-19

**Authors:** Estela Sánchez-Herrero, Mariano Provencio, Atocha Romero

**Affiliations:** Laboratorio de Oncología Molecular, Instituto de Ciencias Biomédicas del Hospital Universitario de Majadahonda, Madrid, Spain; Departamento de Oncología Médica, Hospital Universitario de Puerta de Hierro, C/ Manuel de Falla 1, Majadahonda-Madrid, 28222, Spain

**Keywords:** cinasa del linfoma anaplásico, ADN libre circulante, exosomas, biopsia líquida, cáncer de pulmón de células no pequeñas, inhibidores de la tirosina quinasa

## Abstract

**Introducción:**

Entre el 3 y el 7% de los pacientes con cáncer de pulmón no microcítico (CPNM) presentan reordenamientos genómicos en el gen de la cinasa del linfoma anaplásico (*ALK*). La detección de esta alteración es crucial, ya que los pacientes con CPNM *ALK*- positivos se benefician clínicamente de los inhibidores de *ALK*, mejorando su calidad de vida y supervivencia global (SG), frente a la quimioterapia estándar.

**Contenido:**

En la práctica clínica habitual, las mutaciones de *ALK* se detectan mediante una biopsia de tejido blando. No obstante, la disponibilidad de tejido tumoral se ve comprometida en los pacientes con CPNM a causa de posibles complicaciones quirúrgicas o de la inaccesibilidad del tumor. Además, la calidad y heterogeneidad del ADN pueden dificultar el análisis de las biopsias. Estas limitaciones se pueden superar mediante el uso de las biopsias líquidas, que es un método no invasivo de caracterización molecular del tumor. En el presente artículo, revisamos la tecnología actualmente disponible para la realización de pruebas no invasivas de *ALK* en los pacientes con CPNM, basadas en el análisis del ADN tumoral circulante (ctDNA), ARN tumoral circulante (ctRNA), células tumorales circulantes (CTC), plaquetas educadas por el tumor (TEP) y vesículas extracelulares (VE) como los exosomas.

**Resumen y perspectivas:**

La caracterización molecular no invasiva del tumor es esencial a la hora de mejorar los resultados clínicos y la calidad de vida de los pacientes con CPNM con tumores positivos para la translocación de *ALK*.

## Introducción

El cáncer de pulmón, junto con el cáncer de mama, es el cáncer más frecuente, causante de más de 8,8 millones de muertes al año [1]. El cáncer de pulmón de células no pequeñas (CPNM) es el subtipo de cáncer de pulmón más común, y se suele diagnosticar en estadios avanzados, cuando no es factible un tratamiento curativo [2].

**Figura 1:**
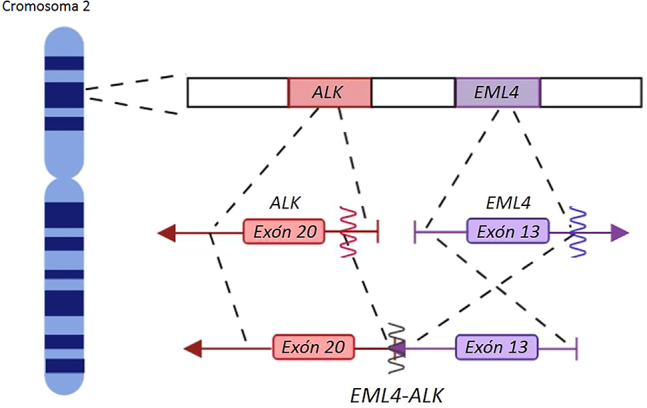
Translocación *EML4-ALK*. Los genes *ALK* (en rojo) y *EML4* (morado) se encuentran en el brazo corto del cromosoma 2 y están orientados en direcciones opuestas. Las flechas indican la orientación del gen. Las translocaciones se producen por inversión paracéntrica [inv(2)(p21p23)], la cual provoca un transcripto de fusión que contiene un dominio catalítico de *ALK* y una mitad amino-terminal de *EML4*. (Figura diseñada por https://app.biorender.com/).

El gen de la cinasa del linfoma anaplásico (*ALK*), descubierto en 1994 en el linfoma anaplásico de células grandes (LACG), codifica un receptor de tirosina quinasa con un dominio transmembrana [3], cuya alteración resulta en la activación constitutiva de *ALK*, generando actividad oncogénica [4], [5]. Se han descrito unas 30 proteínas de fusión de *ALK*. El principal compañero de fusión en los pacientes con CPNM es el gen de la proteína 4 asociada a microtúbulos equinodermos (*EML4-ALK*) [6], [7] (Figura 1), asociado a múltiples variantes de fusión, de los cuales la variante 1 (E13;A20, 33%), la variante 2 (E20;A20, 10%), y las variantes 3 a/b (E6;A20, 29%) son las más comunes [8].

El reordenamiento de *EML4-ALK* se produce en entre el 3% y el 7% de los pacientes con CPNM, lo cual define un subtipo molecular específico de CPNM [9], [10]. Curiosamente, el reordenamiento de *ALK* se observa eminentemente en los pacientes más jóvenes, así como en los no fumadores o fumadores ocasionales con adenocarcinoma.

## Tratamiento del CPNM *ALK*+

**Figura 2:**
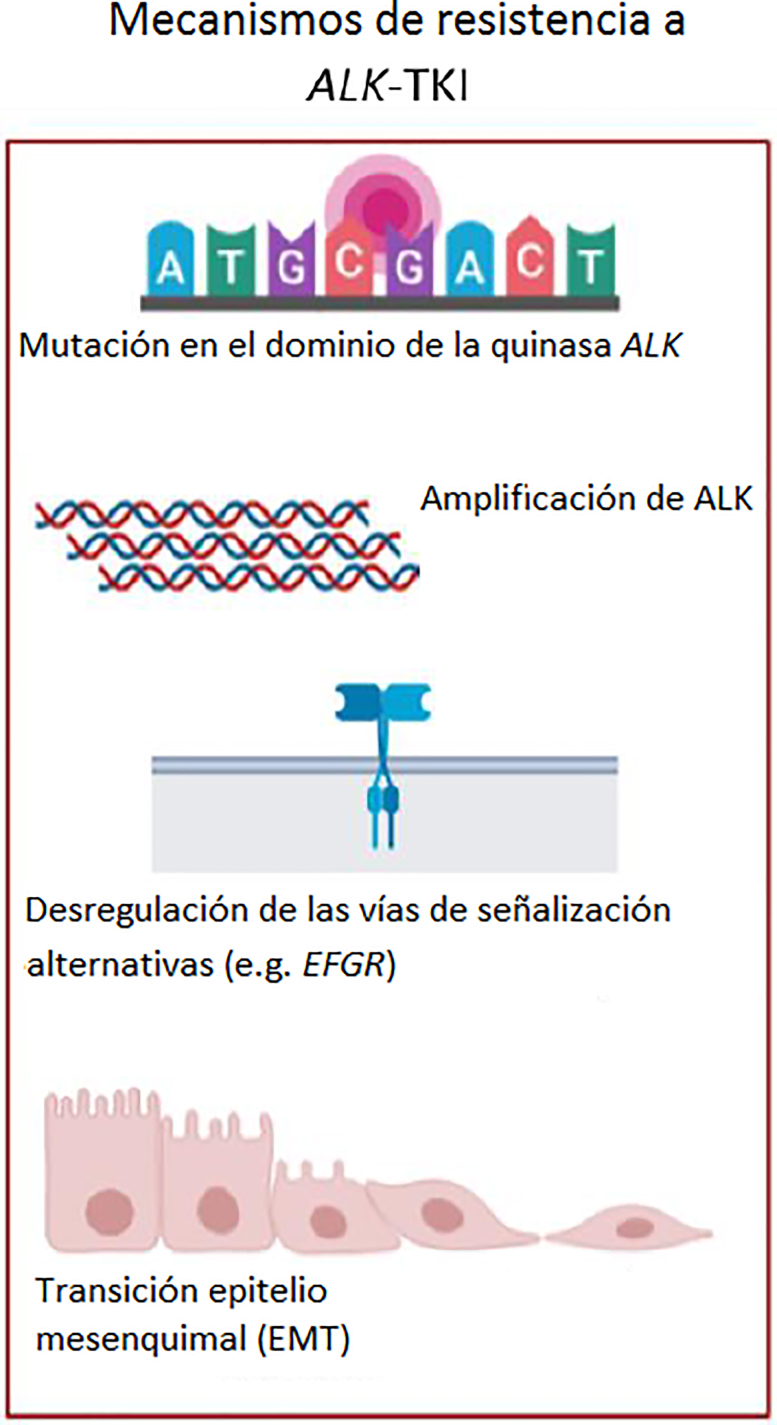
Tratamiento del CPNM positivo para *ALK*. Mecanismos de resistencia a los ITK de *ALK*: Mutaciones del dominio de la cinasa *ALK* (como G1202R, G1269A, F1174L o L1196M), amplificación de *ALK*, desregulación de las vías de señalización “bypass” (como la activación de *EGFR, c-KIT, RAS-MAPK o PI3K-Akt* activation) y la transición epitelio-mesénquima (EMT). (Figura diseñada por https://app.biorender.com/).

El desarrollo de inhibidores de la tirosina quinasa (ITK) contra el gen *ALK* ha mejorado considerablemente los resultados clínicos en cuanto a calidad de vida y pronóstico, habiendo incrementado significativamente la supervivencia libre de progresión (SLP), la supervivencia global (SG) y la tasa de respuesta objetiva (RO), en comparación con la quimioterapia [11], [12]. De este modo, la identificación de pacientes cuyos tumores presentan una translocación de *ALK* es crucial. La National Comprehensive Cancer Network (NCCN) recomienda el uso de crizotinib, un inhibidor análogo de la ATP de *ALK* aprobado por la FDA (Food and Drug Administration) en 2011, como primera opción terapéutica para el CPNM con *EML4-ALK* metastásico o avanzado, con una RO del 74% y una SG de 10,9 meses [11]. Sin embargo, a pesar de su eficacia inicial, aproximadamente el 73% de los pacientes desarrollan resistencia tras 1 o 2 años de tratamiento [13]. No obstante, recientemente han surgido varios inhibidores de *ALK* de segunda generación, como el ceritinib, el alectinib y el brigatinib, que fueron aprobados por la FDA en 2014, 2015 y 2017, respectivamente. Estas terapias mostraron RO y SLP superiores a las obtenidas con quimioterapia de platino con pemetrexed o el crizotinib (RO de 73%, 83% y 71%, y SLP de 16,6 meses, 34,8 meses y no publicada* respectivamente (*seguimiento medio de 11 meses con brigatinib) [14], [15], [16]. Además, los inhibidores de *ALK* de tercera generación como el lorlatinib (un inhibidor selectivo de *ALK* con capacidad para penetrar en el cerebro) fue aprobado en 2018 [17]. Aunque los inhibidores de *ALK* de segunda generación son más potentes y efectivos contra las metástasis en el sistema nervioso central que crizotinib, aún se desconocen los mecanismos de resistencia a estos agentes. Se han descrito varios mecanismos de resistencia a los ITK de *ALK* (Figura 2). Entre ellos se incluyen los mecanismos dependientes de *ALK* como las mutaciones de resistencia en el dominio de la tirosina quinasa de *ALK* (siendo G1202R, G1269A, F1174L y L1196M las más frecuentes); la amplificación del gen de fusión de *ALK*; y los mecanismos independientes de *ALK*, como la desregulación de la vía de señalización *bypass* (activación de *EGFR, c-KIT, RAS-MAPK, PI3K-Akt*, etc.) y la transición epitelio-mesénquima (EMT) [18].

Sin embargo, en la práctica clínica, rara vez se vuelve a realizar una biopsia del tumor debido a la dificultad para acceder al mismo. Así, se desconocen la mayoría de los mecanismos de resistencia a los ITK de *ALK*, lo que resulta en la prescripción empírica de sucesivas líneas de tratamiento, sin conocer el perfil molecular del tumor cuando se produce progresión de la enfermedad. Cabe señalar que las afinidades de unión de los inhibidores de *ALK* están correlacionadas con el tipo de mutación de resistencia. De este modo, existen evidencias preclínicas que sugieren que las mutaciones L1196M o S1206Y confieren resistencia al crizotinib pero no al ceritinib [19]. Es necesario obtener una secuencia de ITK de *ALK* basada en las mutaciones de *ALK* en el momento de la progresión de la enfermedad, para que se puedan personalizar las terapias dirigidas a *ALK*, lo que redundará en mejores resultados clínicos.

## Práctica clínica en los pacientes con *EML4-ALK*

Se pueden emplear diferentes métodos para detectar los reordenamientos de *ALK* en el tejido tumoral. En la práctica clínica habitual, la determinación de la translocación de *EML4-ALK* se realiza mediante hibridación fluorescente *in situ* (FISH), inmunohistoquímica (IHC) o secuenciación de segunda generación (NGS) [20]. Además, cabe señalar que, cuando se identifica una translocación de *EML4-ALK* mediante IHC y/o FISH, no se puede identificar qué variante está presente. Aunque todas las variantes son oncogénicas e inducen dependencia de *ALK* [21], algunos estudios han demostrado que las “oncoproteínas cortas” resultantes de las variantes de fusión de *EML4-ALK* como las variantes 3 y 5 se asocian a peores resultados clínicos [22], [23]. Por otro lado, los productos de las “oncoproteínas largas” de *EML4-ALK* como la variante 2 se asocian a mejores resultados clínicos [24]. Además, cada variante de *EML4-ALK* podría tener diferente sensibilidad a los diferentes ITK de *ALK* actualmente disponibles. Tal es el caso de las mutaciones de *EGFR*, ya que se sabe que los tumores con deleción del exón 19 son más sensibles a los ITK que los tumores que albergan otras alteraciones como las inserciones en el exón 20 [25].

Se suelen emplear métodos como la inmunocitoquímica (ICQ), la reacción inversa en cadena de la polimerasa con transcriptasa inversa (RT-PCR), NGS y los kits comerciales. Por último, nCounter es una nueva tecnología capaz de realizar análisis altamente multiplexados de diferentes moléculas como el ARN, el miARN, las proteínas y el ADN. Con estos métodos se pueden detectar las fusiones de *ALK* empleando diferentes materiales de partida como FFPE, tejidos frescos [16] o tumores derivados de xenoinjertos de pacientes con cáncer de pulmón [26].

No obstante, el diagnóstico basado en la biopsia del tumor presenta algunas limitaciones, como la disponibilidad de muestras para la caracterización molecular del tumor, especialmente en los pacientes con cáncer de pulmón, en el momento de la progresión de la enfermedad. Por lo tanto, la falta de muestras de tejido hace que muchos pacientes con reordenamiento de *ALK* acaben recibiendo quimioterapia en lugar de ITK de *ALK*, con una supervivencia global media desde el diagnóstico de enfermedad metastásica (SG) de alrededor de 12 meses en lugar de 50 meses, como indican varios estudios observacionales en los que se administraron inhibidores de *ALK* [27], [28], [29], [30]. Además, el diagnóstico basado en la caracterización molecular de una sola biopsia puede no reflejar el perfil de la totalidad del tumor, debido a su heterogeneidad [31]. De este modo, es necesario desarrollar nuevos métodos para la identificación no invasiva de la translocación *EML4-ALK*, sus variantes y los mecanismos de resistencia a los ITK de *ALK*.

## Biopsia líquida

**Figura 3:**
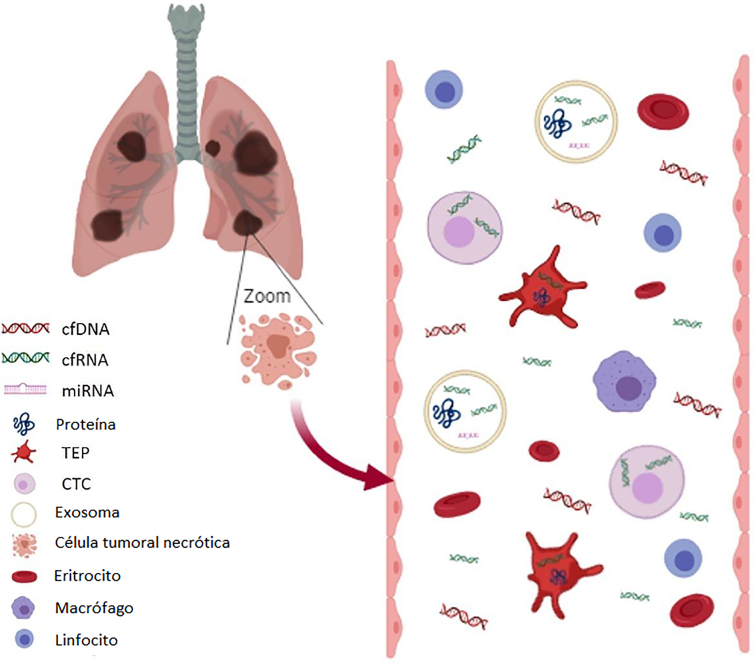
Componentes de las muestras de biopsias líquidas. El cfDNA y cfRNA en sangre proceden de las células moribundas (necrosis o apóptosis). el ADNc es una pequeña fracción de cfDNA. El ADN “wild type” de las células sanguíneas puede diluir el ADNc y, como resultado, las condiciones preanalíticas podrían evitar la lisis celular. Las CTC, plaquetas (TEP) y los exosomas pueden capturar estas y otras moléculas como el miARN o las proteínas de células tumorales. Esta información genética queda mejor protegida en estos compartimentos que en el torrente sanguíneo. (Figura diseñada por https://app.biorender.com/).

El término “biopsia líquida” hace referencia a diferentes técnicas, incluyendo el estudio del ADN tumoral circulante (ctDNA), ARN tumoral circulante (ctRNA), células tumorales circulantes (CTCs), plaquetas plaquetas educadas por el tumor (TEP) y vesículas extracelulares (VE; exosomea, microvesículas, micropartículas, oncosomas) (Figura 3) [32].

La biopsia líquida, un método sensible, seguro y mínimamente invasivo, no presenta las limitaciones de las biopsias de tejido.

Las células tumorales liberan ADN tumoral circulante en el torrente sanguíneo (ctDNA), lo cual se puede objetivar mediante un análisis molecular. Sin embargo, las células sanas también liberan ácidos nucleicos en el torrente sanguíneo, como es el caso de los eritrocitos, los macrófagos o los linfocitos. Por lo tanto, se debe tener especial cautela con las condiciones preanalíticas, con el fin de evitar la contaminación del ADN libre circulante (cfDNA) con ADN de células no tumorales (Figura 3). Algunas técnicas de análisis de cfDNA presentan un índice muy bajo de detección, de 0,1% o incluso inferior, como la reacción en cadena de la polimerasa digital (ddPCR) [33], BEAMing [34], o NGS.

La caracterización del cfDNA resulta útil a la hora de seleccionar los tratamientos moleculares basados en el perfil molecular, la detección temprana de los mecanismos de resistencia [35], [36] y la recidiva tumoral, así como en el seguimiento de la respuesta del tumor a la terapia [37], mediante el empleo de biopsias líquidas secuenciales. Además, se han llevado a cabo varios estudios para comparar la detección de la translocación *EML4- ALK* en tejido fijado en formalina e incluido en parafina (FFPE), frente a la biopsia líquida que han demostrado que se puede utilizar cfDNA como sustituto del ADN de tejido FFPE [38].

Sin embargo, la comprobación de la fusión de *ALK* mediante biopsias líquidas es un proceso complejo. En primer lugar, identificar el reordenamiento genómico empleando cfDNA es difícil, ya que se suele desconocer cuáles son los puntos de ruptura génica *(breakpoints)*, y los reordenamientos suelen implicar un gran número de pares de bases. Aunque la detección de transcriptos de fusión es más sencilla, requiere el uso de cfRNA que, a diferencia del cfDNA, se degrada muy rápidamente debido a la presencia de ribonucleasas en el torrente sanguíneo. Por lo tanto, el análisis del ARN contenido en vesículas como los exosomas o las plaquetas, donde está protegido de las ribonucleasas, puede ser una estrategia efectiva de análisis.

## Estrategias de detección de *EML4-ALK* mediante biopsia líquida

### ADN libre circulante (cfDNA)

Según recomendaciones recientes, los paneles de NGS de biopsias líquidas basados en cfDNA son útiles a la hora de detectar a los pacientes con CPNM con *EML4-ALK* con mutaciones de resistencia, cuando no se puede obtener una nueva biopsia del punto de progresión [19]. NGS parece ser el mejor método, ya que presenta niveles de sensibilidad y especifidad del 80 y el 100% respectivamente [39]. NGS puede aportar información no sólo sobre las mutaciones de resistencia de *ALK*, sino también sobre otros mecanismos de resistencia molecular que se pueden emplear como diana terapéutica en un ensayo clínico, o ser expandidos para los estudios con fármacos. Sin embargo, la aplicación generalizada de NGS de biopsias líquidas se ve limitada por la necesidad de contar con un equipo específico, así como por su elevado coste.

Por otro lado, resulta difícil detectar en cfDNA alteraciones como la translocación *EML4-ALK*, ya que se suelen desconocer los puntos de ruptura a nivel de ADN. Además, todas estas alteraciones implican un elevado número de pares de bases y el cfDNA está fragmentado (suele tener un pico de aproximadamente 150 bp). A diferencia de las mutaciones en *EGFR*, la detección del reordenamiento de *ALK* mediante el análisis de cfDNA apenas se utiliza en la práctica oncológica.

A pesar de todo lo anterior, se han detectado fusiones génicas de *EML4-ALK* mediante el análisis de cfDNA con análisis NGS de biopsias líquidas diseñados para detectar uniones en puntos de ruptura. Este método de análisis ha sido validado mediante el empleo de líneas celulares personalizadas con 10 fusiones génicas de *EML4-ALK* y 26 fusiones sintéticas diseñadas para aplicar el análisis InVisionFirst [40]. Además, se ha analizado el estado de fusión de *ALK* en cfDNA plasmático de pacientes con CPNM empleando NGS basada en la captura, con una especifidad del 100% [41]. Esto indica que se pueden detectar reordenamientos mediante la secuenciación de cfDNA. No obstante, debido a las limitaciones anteriormente descritas, los resultados negativos deben interpretarse con cautela.

### ADN libre circulante (cfDNA)

Las aberraciones complejas como los reordenamientos genómicos extensos, incluyendo las translocaciones, se pueden detectar fácilmente como transcriptos de fusión a nivel de ARN. No obstante, a diferencia del cfDNA, el cfRNA se degrada rápidamente, lo que supone una importante limitación. Existen evidencias de que la optimización de las condiciones preanalíticas de las muestras de biopsias líquidas pueden mejorar la sensibilidad de RT-PCR basada en el cfRNA para la detección de transcriptos de fusión de *EML4-ALK* [42].

Parte del cfRNA presente en el torrente sanguíneo es capturado en diversos compartimentos donde está más protegido y funcionalmente activo [43]. Actualmente, los compartimentos más frecuentemente estudiados incluyen las células tumorales circulantes (CTC), las plaquetas plaquetas educadas por el tumor (TEP) y las vesículas extracelulares (VE, exosomas, microvesículas, micropartículas, oncosomas).

### Células tumorales circulantes (CTC)

Las células tumorales circulantes (CTC) son células circulantes presentes en el torrente sanguíneo de los pacientes oncológicos liberadas por un tumor primario. Las CTC están implicadas en la metástasis, ya que tienen capacidad para adherirse a las paredes de los capilares y penetrar otros tejidos [44]. Algunos estudios indican que la presencia y elevados niveles de CTC están asociados a un peor pronóstico [45], [46]. Sin embargo, las CTC tan solo representan una pequeña fracción de la población celular de la sangre periférica en los pacientes oncológicos, tanto en términos absolutos (<10 cell/mL), como relativos, en comparación con otras células sanguíneas (1 CTC por cada 10^6^–10^7^ leucocitos) [47]. Por lo tanto, el empleo de CTC para la detección de reordenamientos de *EML4-ALK* debe estar basada en estrategias de detección muy eficaces. Del mismo modo, debido a la heterogeneidad de los tumores, las CTC pueden presentar variabilidad genómica, lo cual debe tenerse en cuenta [48]. Así mismo, las CTC pueden presentar diferencias con respecto a los tumores primarios y metastásicos [49].

Aparte de estas limitaciones, se han detectado reordenamientos de *EML4-ALK* en CTC de sangre periférica en pacientes con CPNM [45], [46], [47]. Se han desarrollado diferentes métodos para aislar e identificar CTC. Sin embargo, el único método aprobado por la FDA para la determinación de CTC en sangre es el sistema CELLSEARCH® [44]. Otros métodos empleados para detectar reordenamientos de *ALK* implican un proceso de enriquecimiento previo a la detección de las CTC, o vice versa, con el fin de aumentar su sensibilidad y especifidad [45].

La tecnología basada en el tamaño de las células tumorales epiteliales (ISET) es otro método para aislar las CTC que ha mostrado ser superior al sistema CELLSEARCH® system [50]. Si se realizan secuencialmente ISET, FISH y IHQ, las CTC demuestran ser una herramienta fiable para la detección de reordenamientos de *ALK* en pacientes con cáncer de pulmón, con una concordancia del 90% con respecto a las biopsias de tejido [51]. Además, se han detectado mutaciones de resistencia del gen *ALK* como L1196M en CTC [52] y se pueden expandir ex vivo para probar fármacos. Por lo tanto, esta fuente no solo tiene utilidad clínica para el diagnóstico, sino también como una herramienta potencial para determinar la sensibilidad al fármaco, así como en la medicina de precisión personalizada.

### Plaquetas educadas por el tumor (TEP)

Las plaquetas son fragmentos celulares sin núcleo producidas por los megacariocitos en la médula ósea. Estos componentes de la sangre pueden secuestrar ARN tumoral mediante un mecanismo dependiente de las microvesículas, causando alteraciones en el ARN y en el contenido proteico [53]. Como resultado, estas plaquetas con formación tumoral (TEP) tienen una función alterada, pudiendo estimular la supervivencia de las células tumorales y la metástasis [53]. Por otro lado, recientemente se ha publicado que las plaquetas están implicadas en la respuesta inmune y en las enfermedades pulmonares inflamatorias [54]. Además del contenido de plaquetas, así como su número y tamaño, en el diagnóstico y pronóstico del cáncer también se utilizan marcadores proteicos como la Pselectin [55]. Sin embargo, los ensayos basados en las plaquetas presentan algunas limitaciones como la reducción del número o su posible activación con algunas terapias, lo cual puede afectar a la interpretación de los resultados.

Se han identificado reordenamientos de *EML4-ALK* mediante el análisis de ARN plaquetario de pacientes con CPNM previo a la administración de una terapia con ITK de ALK en el momento de la progresión de la enfermedad. Así mismo, han reaparecido reordenamientos de *EML4-ALK* incluso antes de que se demostrara progresión de la enfermedad mediante PET-TAC [56]. Por otro lado, cuando los pacientes respondían a terapia, no se detectaba la translocación de *EML4-ALK* en las plaquetas. La sensibilidad y especifidad para la detección de reordenamientos de *EML4-ALK* en ARN plaquetario oscilan entre el 65% y el 100%, respectivamente [56]. Aun cuando se procesan las muestras de biopsia líquida en condiciones preanalíticas óptimas, la sensibilidad de cfRNA para la detección de reordenamientos de *EML4-ALK* fue inferior a la del ARN plaquetario (21% y 65% respectivamente) [36].

Las plaquetas también pueden capturar vesículas extracelulares (VE) liberadas por las células tumorales con ARN tumoral [53] como aquellas que contienen el gen de fusión de *EML4-ALK*. Las VE son otro material de partida para el aislamiento de ácidos nucleicos y se emplean para detectar la translocación *EML4-ALK* en las muestras de biopsia líquida.

## Exosomas

Los exosomas son un tipo de VE son nanovesículas (30–200 nm) liberdas tras la fusión de cuerpos mutivesiculares con la membrana plasmática al final de la vía de reciclaje endocítico. Las células tumorales liberan exosomas con cambios significativos en su composición, que pueden actuar como vehículo para el intercambio de material genético y proteico entre células. Esto provoca modificaciones como la angiogénesis, el desarrollo de resistencia terapéutica, metástasis y una mayor proliferación [57].

Muchos de los posibles biomarcadores no invasivos que se ha estudiado en biopsias líquidas se encuentran en los exosomas [58], [59]. Los biomarcadores exosómicos pueden tener mayor valor diagnóstico y pronóstico que el cfDNA [60].

No solo se han aislado exosomas en sangre, también en otros fluidos biológicos como la orina. Sin embargo, la concentración de VE es inferior en orina que en sangre [48]. Se han descrito diferentes métodos para aislar exosomas, siendo la ultracentrifugación y algunos kits comerciales los más comunes. Por otra parte, estas técnicas tienen un rendimiento diferente a la hora de detectar exosomas, y aportan diferentes niveles de pureza. De este modo, la ultracentrifugación tiene un rendimiento inferior, aunque proporciona exosomas de mayor pureza, mientras que los kits comerciales dan exosomas de inferior pureza con un mayor rendimiento [61], [62]. Sin embargo, la ultracentrifugación es un método complejo. Las nuevas tecnologías como los kits comerciales o los métodos de NGS basados en la captura en lugar de la amplificación podrían tener mayor sensibilidad a la hora de detectar las fusiones de *ALK* [63], [64]. Además, estas nuevas tecnologías pueden ser más sencillas y rápidas y podrían ser implementadas en la práctica clínica habitual.

Se han realizado pocos estudios para analizar genes de fusión en exosomas. Sin embargo, se ha detectado la translocación *EML4-ALK* mediante el análisis de ARN exosómico aislado en plasma de pacientes con CPNM, con una especifidad del 100% y una sensibilidad del 64–70% comparado con el análisis de tejido [63], [65].

## Enfoques futuros

Varios estudios han mostrado una excelente especifidad para la detección de *EML4-ALK* basada en el uso de diferentes materiales de partida para aislar ácidos nucleicos en biopsias líquidas, considerando la biopsia de tejido como la piedra angular. Su sensibilidad, sin embargo, es bastante baja, y los resultados negativos han de ser interpretados con cautela [41], [45], [56], [63]. Por lo tanto, el reto es hallar la forma de mejorar la sensibilidad de la biopsia líquida para detectar reordenamientos de *ALK* y mejorar las tecnologías de aislamiento y detección para establecer protocolos sólidos y reproducibles. Los métodos basados en PCR, así como las metodologías dPCR, BeAMing, nCounter y NGS poseen una mayor sensibilidad para la detección de reordenamientos de *ALK* con respecto a otras estrategias, aunque presentan algunas limitaciones.

Finalmente, es preciso realizar estudios prospectivos de cohorte para evaluar la precisión diagnóstica de los métodos basados en biopsia líquida para la detección de *EML4-ALK*.

## Conclusiones

La biopsia líquida es un método no invasivo que puede mejorar la detección de translocaciones de *EML4-ALK* y la identificación de los mecanismos de resistencia a los TKI de *ALK*. El empleo de biopsias líquidas podría facilitar el diagnóstico temprano de CPNM *ALK* positivo y mejorar los resultados clínicos, lo que conlleva un mejor pronóstico y calidad de vida para los pacientes con CPNM *ALK* positivo.
